# Hospital and Institutional News

**Published:** 1917-04-14

**Authors:** 


### HOSPITAL AND INSTITUTIONAL NEWS.

### WHAT IS TRUTH?

We have now received from a firm of Solicitors a complaint that the Article published under this heading in our issue of March 17 was a very serious libel on clients of theirs, charging them in effect with "conspiring with *Truth* to foist upon the "public a perfectly worthless system of memory-"training without the slightest foundation for "making such charges, and which if allowed to go "unrefuted would have most serious consequences "to our Clients." We do not ourselves consider that the article imputes conspiracy of any sort, and we disclaim any intention to make such an imputation. Nor do we find in it any statement or innuendo that the system referred to in the Solicitors' letter is perfectly worthless. It is manifest from the published testimonials that many persons have obtained much benefit from it. But to develop "a Never Failing Memory" (see *Daily Mail* Advertisement), or "a Never Forgetting Memory" (see *Times* Advertisement) is a pitch of success which we conceive to be unattainable by or through any system, however perfect or successful. If, however, the Article has been read to convey such an imputation as the Solicitors define we at once disavow any such intention, and withdraw the words complained of with an expression of our regret that the Article has been so misinterpreted, and our apologies to the Clients for the annoyance caused to them by its publication.

### AN IMPORTANT POST UNFILLED.

The Royal Infirmary, Bristol, has decided not to fill the office of president, which became vacant on the death of Sir George White, until the end of the war. The reason for this course is that anyone whom the committee wished to invite to fill the post would be too much occupied to be able to give the necessary time and attention to the work. From this it may be inferred that the committee have virtually decided on the person to whom they will offer the position. It requires no reminder that the late Sir George White was a busy, enthusiastic, bustling man who spent much energy and time in raising the £50,000 which paid off a heavy overdraft and helped to build the handsome surgical block, which was opened by the King and Queen, and is now, as originally planned in case of war, being used as a military hospital. In order to carry out his original scheme Sir George White determined on the sale of securities. which was a thorough one, and acted on the hope of being able to replace the income from capital thus expended by a vast list of annual subscribers. It was a difficult, and indeed somewhat speculative, endeavour. The position to-day is therefore worth recording as the sign of his measure of success. When he became treasurer in 1904 the adverse balance on the ordinary income (£11,792) was £4,700. To-day, with an income of £15,472, the excess of expenditure is £8,504. The Royal Infirmary, Bristol, has a fair revenue from annual subscribers, but there are hardly enough of them to fulfil his dream of making the hospital self-supporting mainly through their means. A big stroke of luck occurred when the Royal Infirmary received its share of a very large estate. But Sir George's financial policy has an unusual interest, as experiments of this kind are not very common.

### PROPOSED LARGE GIFT OF LAND TO NEWCASTLE ROYAL INFIRMARY.

Next week the Newcastle City Council will be recommended by the Town Moor and Parks Committee to give to the Newcastle Royal Infirmary the freehold of fourteen and a half acres on the Newcastle Leazes for the building of the considerable extensions that the hospital now requires. It is hoped that the Freemen may agree to surrender their herbage rights, and if the City Council agree to make the grant the infirmary authorities have undertaken to carry out the wishes of the Town Moor Management Committee in regard to the planting and arrangement of the ground, and further to promote a Parliamentary Bill for its appropriation. Of the needs of the infirmary there is no question, and in view of the work it has carried out since the war there should be little doubt of the City Council's decision.

### DEVELOPMENTS AT WARRINGTON INFIRMARY.

Mr. J. H. Smethurst, chairman of the board of Warrington Infirmary, made, as usual, some good points in his recent speech, in which he appealed for £4,000 to meet the future needs of the hospital. Since it is one of those which works harmoniously on special matters with the Corporation, its work for school children and for the Corporation's scheme for the treatment of convalescents is likely to increase. The Corporation is ready to recognise the value of the hospital's work, and a scheme satisfactory to both will probably be arranged. In dealing with the deficit of £2,600, Mr. Smethurst said that the assets of a voluntary hospital were the value of its work and the public confidence which was based on it, so that he was not depressed as he would have been had he been applying the canons of commerce to its balance-sheet. Mr. Smethurst provided a good send-off for his appeal by producing a precious promise of £250 from an old supporter of the hospital, and the committee, we may add, agreed to an alteration of the rules by which two representatives of the ladies' committee will have seats on the board. This number seemed to some "miserably small," but the difficulty of representation is to avoid defeating its object through the creation of an unwieldy body, which does not even represent the good sense of its individual members.

### THE SANITATION OF OSBORNE.

An animated discussion has been proceeding lately in the columns of the leading daily paper as to the cause of the severe and serious epidemics of zymotic disease at Osborne. This school for the Navy has recently suffered from a severe visitation, in which there have been several fatalities; and the allegation is roundly put forward that there are fundamental defects in construction, administration, and supervision, to which these recurring epidemics, with the loss of precious young lives, are due. Although some of those who have joined in the correspondence deny that Osborne suffers more heavily than other schools containing large numbers of boys at the most susceptible ages, there is yet sufficient definiteness about the complaints to render them, in our judgment, worthy of expert investigation: not by a jury of matrons and parents, as one indignant parent suggested, but by professional hygienists. The complaints which require most investigation are four in number. One is, that the buildings are of faulty design or construction, and hence insanitary. The next is that they are grossly overcrowded, so that epidemics are much more likely to spread and to take serious forms. The third is that the cadets are systematically overworked. And the last is that until they actually fall sick they are attended by ex-sailors, whose knowledge of the prevention and detection of disease is as rudimentary as their practice of hygienic principles is likely to be imperfect.

### RESEARCH INTO REFERRED PAIN.

The energy and journalistic ability of the medical correspondent of the *Times* have been many times conspicuous in the last year or two, though a good many of his *ex-cathedra* utterances have been quite contrary to the general consensus of opinion in the medical profession. His right to speak for that profession and his depth of knowledge of medicine are not likely to receive enhanced recognition if he writes many more articles similar to that which he contributed to the *Times* of April 5 on "Pain and Disease." In that article he describes as brand new certain researches upon referred pain in appendicitis and other diseases which—if his description of them is correct—are neither new nor particularly valuable. It is true that he mentions some ideas on the point under consideration which were propounded by Sir James Mackenzie in 1891; but he entirely ignores the researches of Dr. Henry Head not very long after, of Mr. James Sherren about 1903, and of several subsequent observers who have very largely anticipated the work now described as new. His statements convey the impression that by observations of skin tenderness the diagnosis of appendicitis and of diseases of other organs can be rigidly confirmed or excluded. That view is not new by a good many years, nor has it been found to be accurate. The question of any one journalist's reliability may seem of no account in the eyes of Fleet Street; but it is a pity for a medical man occupying the unique platform of *Times* correspondent to fall below the best standards of his own profession.

### MAKING THE WOUNDED A BONE OF CONTENTION.

Misunderstandings between Boards of Guardians and voluntary hospital managers recur regularly, if rarely, because a want of tact on one side or the other leads to the suspicion that the hospitals or the Guardians are not doing their duty by the poor. Guardians resent having cases turned over to them from a voluntary hospital even when the hospital is full, although they can hardly deny that many patients whose technical destitution makes them proper subjects for treatment at a Poor-Law infirmary are in fact very often accepted at a hospital's doors. So far, however, the reception of wounded has caused no friction, but this happy record is now broken by the Portsmouth Guardians, who have attacked the committee of the Royal Portsmouth Hospital on the ground that certain cases had been sent to the Poor-Law infirmary because, if you please, the hospital beds were full and tenanted by many of the wounded. We have too often reminded hospitals of their duty to the civil population to incur the charge of blinking the facts in this case. But the Guardians have put themselves in the wrong by alleging that the hospital has been taking wounded for the sake of profit. That is a venomous suggestion, and we have only heard of one hospital, and that the poorest in London, which ever has claimed any profit from the Government grant. To allege a pecuniary motive is insulting and uncalled for in the case of a big institution like the Royal Portsmouth Hospital. It is the Government, not the hospitals, which name the number of beds to be reserved for the wounded, and though hospitals should not do more than the civil claims upon them justify, they may look for co-operation and not abuse in their endeavour, with the Guardians, to shoulder the double burden imposed on them by the war.

### ANOTHER HOSPITAL FOR SHELL-SHOCK CASES.

In connection with the Hospital for Epilepsy and Paralysis in Maida Vale a branch hospital of one hundred beds is nearly ready for opening. The object of the new institution is to treat and "re-educate" soldiers who have become victims of shell shock and grave nervous disturbance. This, of course, will not be the first special institution of this kind, but since, on the face of it, the primary needs are absolute quiet, seclusion, and such restful influences as the world of Nature provides, it is clear that an already busy London hospital is not in some respects the best place for such cases. The statistics of success and failure with this class of patient will be interesting when they become due, and perhaps give a more solid groundwork than we now have to the nature of neurasthenia and the best methods of treating it.

### REAPING THE TARES.

Our readers will not be surprised to learn that the criticism to which the Merthyr General Hospital at once lay open for the decision to close beds has been promptly uttered by the very workmen whose position on the board has been part of the recent difficulties. Thus at a recent mass meeting of the Merthyr Trades and Labour Council, in addition to the reiteration of demands for further representation, a resolution was carried which protested not only against the board's opposition to the workers' demands, but also against its "drastic action in closing beds when money was available."

These last words are significant, and show, as we have so often explained, that the first thing which a hospital loses when it closes beds is public confidence, whereby its support is enfeebled and its influence imperilled. The board should undertake to reopen the beds, the workmen to guarantee a reasonable sum for their support, and both sides can then discuss the question of representation in the light of mutual co-operation. The principle, once again, is that donors are accorded representative rights, but that the object of all donations is not to purchase seats on a hospital board, but to secure the treatment of sick people in need as an act, not of bargaining, but of charity. This is the only way in which an unfortunate situation, badly handled, can avoid the trouble which it invited.

### AN UPHILL FIGHT IN KENSINGTON.

Interested observers of hospital affairs will let their attention linger for a moment when the annual report of the Kensington and Fulham General Hospital comes round. In spite of what might seem the insuperable difficulties of its origin, its mere appearance, and its situation and site, this remarkable institution presses up the Hill Difficulty as persistently as ever. Encouragement has been small, but it has come, like recognition, in the end from the high authorities. The King's Fund raised its grant from £25 to £50 last year. Lord Claud Hamilton remains president, which must be regarded as a proof of his belief in the hospital's future. Since its new start a few years back we have shared this belief, but, in a real and no ironic sense, its official positions are those which ambitious men would be most inclined to take, since only very hard work can raise the hospital to the position and importance to which its friends naturally look forward. It is encouraging, therefore, to see that last year the income showed an increase, that the reception of soldiers added public interest as well as income to its credit, and that the new workers whom it is attracting seem to be no less energetic and enthusiastic than the old.

### GERMAN WOMEN OF THE RED CROSS.

A terrible story of German brutality to British wounded on a grand scale has reached England from a correspondent of the *Times* in Switzerland. To German brutality the world is getting accustomed—almost inured; but it has been hoped and believed in many quarters that the official brutality of Germany's rulers and of German officers was not reflected to nearly so great an exent in the behaviour of individual German workers and women. According to the ghastly records disclosed from Switzerland, it seems as if not even hospital nurses and other Red Cross workers in Germany have escaped the contamination of Prussian savagery. For many incidents of deliberate cruelty to wounded and starving English prisoners are narrated; and it is roundly asserted that these are not isolated cases, but merely instances selected out of hundreds. What the revelations will be when our men who are still imprisoned in Germany get home we shudder to think of. To go into details of these horribly distressing incidents here is unnecessary; they stand in last Wednesday's *Times* for all to read. But it may perhaps be worth while to adopt the concluding phrases of a remarkable contribution. "The German Red Cross has prostituted the sacred sign and shamed its name. It has forfeited all right to be considered as an organisation of humanity. It is an organisation of German militarism, and of that only; and it has dragged the Red Cross flag and the reputation of German womanhood in the mud." And next below the article in question is a report—may it prove unfounded—that British prisoners are being systematically butchered in Germany; we wish we could say that the thing is impossible.

### THE WOMAN HOUSE SURGEON AFTER THE WAR.

It is impossible to glance over the annual reports of hospitals this year without noticing frequent expressions of gratitude for the services of women house surgeons. These, to say the least, sound no more formal than the customary acknowledgments paid to their male predecessors, and it is sometimes argued that the war has shown how fit are women to hold posts upon a hospital's honorary staff. We do not dispute this view, but it is perhaps well to remind enthusiasts that only the recurrence of peace can test the regard which women doctors have really gained. We shall not be surprised if many hospitals prefer to retain the services of women in these positions. But it is too early to say, and the issue is ultimately dependent on circumstances which have no immediate relation to the value of their work during the hard times through which we have all been passing.

### THE CAMPAIGN AGAINST VENEREAL DISEASES

The Local Government Board has informed the Portsmouth Town Council that it is disposed to consider that the Council might with advantage make use of a proposed home in connection with the scheme for the treatment of venereal diseases. This permission should prove of the utmost value, for it obviously will be extended to all other authorities. The idea in Portsmouth is to establish, in conjunction with the local Church Council, a hostel or rescue home so as to enable girls to be removed from evil surroundings while they are being treated for the disease. The scheme will be supported out of the rates, and the Local Government Board will repay 75 per cent. of the cost.

### RATEPAYERS ONLY MAY COMPETE AT DERBY.

Local architects came in for rather severe criticism at the last meeting of the Derby Board of Guardians, when a discussion took place regarding plans for the new workhouse infirmary, which is estimated to cost £50,000. It was recommended that the services of a certain professional gentleman should be secured for the preparation of the plans, etc., but several members raised objection to the course of action suggested, on the ground that it would be fairer to invite competitive plans, thus giving other capable architects the opportunity of obtaining the commission. Canon Browne remarked, "You prate about the super-excellence of

Derby architects, but there are precious few monuments to it in the architecture of the town." It was pointed out by the clerk to the Board (Mr. R. Grantham) that competitive plans would entail an expenditure of from £200 to £300 in fees, and might result in delaying the work. The Board, by twenty votes to fourteen, resolved to invite plans from ratepaying architects only.

### BRADFORD GUARDIANS ON HOSPITAL ARCHITECTURE.

An interesting discussion took place at the last meeting of the Bradford Board of Guardians on the changes that have come about in relation to the type of building most suitable for hospital purposes. Medical authorities, it was remarked, have now completely revised their opinion as to the style of building required. Mr. Rawdon remarked that the time for erecting barrack buildings has gone by, and it was agreed on all hands that for hospital purposes single-decker or two-decker buildings are much better. The opinion was expressed that in premises constructed on these new lines better lighting and better ventilation can be provided. The only reason, we take it, for departing from this type of construction is the inordinate cost of land in large towns, a difficulty which always confronts the architect of an urban hospital. This point seems to have been overlooked in the discussion just alluded to.

### FORESIGHT AT THE RETFORD HOSPITAL.

In view of the additional demands on the accommodation which will follow the new colliery developments in the district, it is almost a foregone conclusion that there will have to be an extension of the Retford Hospital. The matter has already been taken up by the governors, and the possible requirements in the way of new buildings are to be thoroughly gone into at the next monthly meeting in May. The hospital, by the way, has just received a donation of £100 from Mr. W. W. Cranfield.

### CURIOUS PROPOSAL BY AUSTRALIAN SATURDAY FUND.

The Hospital Saturday Fund of Sydney has made a novel proposal to the Board of Directors of the Royal Prince Alfred Hospital, which amounts practically to a proposal that the Saturday Fund shall raise all funds subscribed by the public in any form, not merely those gathered in by joint effort of the hospitals and the Fund. Thus, in addition to the house-to-house, street, and industrial collections, the Saturday Fund would take over from the hospitals the collections of the separate amounts now obtained by them from individual subscribers. The executive of the Prince Alfred Hospital, the chief institution concerned, has not agreed to the scheme. This is not surprising; what was suggested was something quite unknown in the history of hospitals anywhere, and involved the handing over to an outside body of the raising of funds on a general impersonal plan for hospitals which had built up for themselves a *clientèle*, as it were, of subscribers, some dating back nearly fifty years, who had in many cases given sums for strong personal reasons or out of sympathy for the institution. The *Prince Alfred Hospital Gazette*, in reviewing fully the proposals, expresses the opinion that probably the total result of the change would be that instead of an increase upon the present amounts received by joint hospitals, the total would be very considerably less. Fifty years' experience confirms the probable accuracy of this contention.

### THE RATIONING PROBLEM IN HOSPITALS.

Food grumbles are to be heard in not a few hospitals nowadays, though it is not often that they crystallise into such a definite complaint as was made recently in regard to the rations issued in the Kilton Hill Hospital, Worksop. The allegation, however, has been investigated and found groundless. At the last meeting of the Worksop Board of Guardians, the clerk read a letter from Col. Vincent, who said that in company with a quartermaster he visited the hospital for the purpose of inquiring into the complaint that there was a shortage of food, and he had satisfied himself that the complaint was unfounded, and the quantities of the rations issued were in every way sufficient. The Guardians, at the same meeting, gratefully accepted an offer by the Duke of Portland of a ton of potatoes for the disabled soldiers at the Kilton Hill Hospital.

### MEMORIALS.

As memorials to their late presidents, Mr. Ben Pickard, M.P., and Mr. Enoch Edwards, M.P., the Miners' Federation of Great Britain has decided to endow a bed at the Beckett Hospital, Barnsley, in memory of Mr. Pickard, who resided in that borough, and at the North Stafford Hospital, Burslem, in memory of Mr. Edwards, who was a Bursiem man. The endowment will cost £1,250 in each case. Marble busts of the two miners' leaders are also to be erected in the institutions.

### THIS WEEK'S DRUG MARKET.

Business in the drug market had been suspended during the greater part of the period that has elapsed since the last report appeared, but was resumed immediately after the holidays. The upward movement of prices is likely to continue, and the difficulty of obtaining supplies of some of the commonly prescribed drugs is likely to become more acute. Epsom salts is again dearer owing to continued shortage. Opium has advanced in price still further, and under the circumstances a rise in makers' quotations for morphine would certainly be justified. The value of quinine is not quite so firmly maintained, but any spurt in the demand would no doubt send the price up again. In synthetic drugs there have been no changes of importance, but the slightly downward tendency in the price of aspirin is still noticeable. The advanced price of strychnine is well maintained. Citric acid, tartaric acid, and cream of tartar are all tending upwards in price. Supplies of cocaine are still insufficient, and the price is maintained. Gamboge and gum guaiacum continue scarce. The quantities of glycerophosphates, hypophosphites, and magnesia available are insufficient.

